# Peptide-Modulated Activity Enhancement of Acidic Protease Cathepsin E at Neutral pH

**DOI:** 10.1155/2012/316432

**Published:** 2012-12-17

**Authors:** Masayuki Komatsu, Madhu Biyani, Sunita Ghimire Gautam, Koichi Nishigaki

**Affiliations:** ^1^Department of Functional Materials Science, Graduate School of Science and Engineering, Saitama University, 255 Shimo-okubo, Sakura-ku, Saitama-shi, Saitama 338-8570, Japan; ^2^Rational Evolutionary Design of Advanced Biomolecules, Saitama (REDS), Saitama Small Enterprise Promotion Corporation, No. 552, Saitama Industrial Technology Center, 3-12-18 Kami-Aoki, Kawaguchi, Saitama 333-0844, Japan

## Abstract

Enzymes are regulated by their activation and inhibition. Enzyme activators can often be effective tools for scientific and medical purposes, although they are more difficult to obtain than inhibitors. Here, using the paired peptide method, we report on protease-cathepsin-E-activating peptides that are obtained at neutral pH. These selected peptides also underwent molecular evolution, after which their cathepsin E activation capability improved. Thus, the activators we obtained could enhance cathepsin-E-induced cancer cell apoptosis, which indicated their potential as cancer drug precursors.

## 1. Introduction

Although a number of enzyme inhibitors, such as kinase inhibitors and protease inhibitors, have been successfully used in cancer therapy, very few enzyme activators have been successfully applied [1–3]. This discrepancy is partly because enzyme activators are difficult to identify as there are currently no rational design principles or effective screening methods that can be used [4]. Because various diseases are caused by reducing the activities of endogenous enzymes [5, 6], a general method for identifying enzyme activators is highly desirable. In particular, a method for obtaining enzyme-activating peptides is attractive because of the potential of activators to reveal phenomena that cannot be elucidated by inhibitors [7].

A preliminary approach used an *in vitro* evolution method called the evolutionary rapid panning system (eRAPANSY) [8], and peptides that moderately enhanced cathepsin E activity were successfully identified after secondary library selection [9]. Cathepsin E, which usually operates at acidic pH [10], has been shown to induce cancer cell apoptosis [11, 12] and inhibit tumor angiogenesis [13] at neutral pH, which promotes the finding of its activators for cancer therapy. Therefore, in this study, we sought to obtain peptides with sufficiently high biological activity that would be suitable for medical purposes. 

To achieve this, we adopted the systemic *in vitro* evolution method (eRAPANSY) along with the paired peptide method (PPM), in which selected peptides were arbitrarily combined by linking through a definite length of a spacer sequence. This resulted in a paired peptide library containing a set of peptides consisting of two peptide moieties, each of which was per se functional. Thus, linking of these peptide aptamers via a spacer is highly promising for obtaining far more advanced peptides. This strategy has already been successfully applied to obtain cathepsin E-inhibitory peptides. Thus, this study confirms the effectiveness of systematic *in vitro* evolution combined with the progressive library method. 

The paired peptide library was subjected to a conventional cDNA display [14] and function-based selection could identify cathepsin E-activating peptides with greater activating capability. We also examined the biological activities of these peptides by examining the induction of cancer cell apoptosis. This showed that these peptides were biologically active.

## 2. Materials and Methods 

### 2.1. Preparation of Cathepsin E and Its Substrate

Cathepsin E was isolated from rat spleens as previously described [15]. The fluorogenic substrate for cathepsin E at neutral pH (pH 7.4) was the same as previously reported [14]. The fluorogenic substrate (10 mM) was prepared by dissolving in 100% DMSO and diluting in reaction buffer immediately before use.

### 2.2. Construction of the Paired Peptide Library

**Figure 1:**
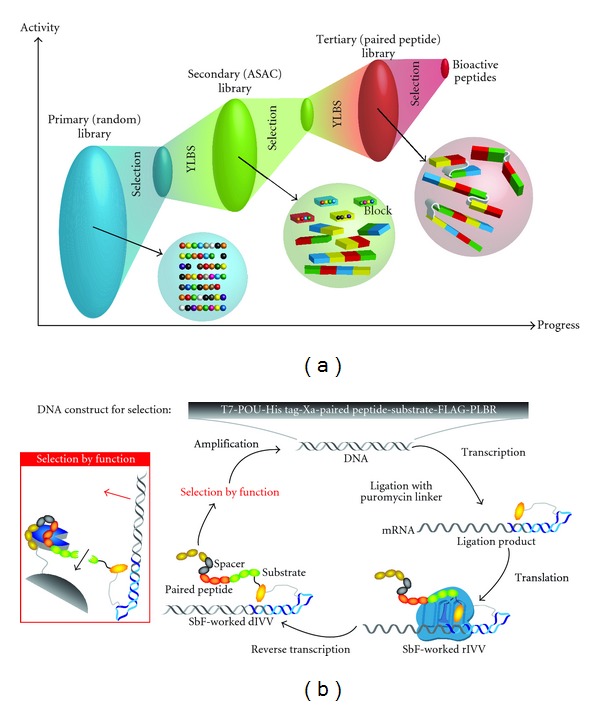
Systematic of the *in vitro* evolution strategy for obtaining enzyme activators. (a) Progressive library. Functional peptides were identified from the first random peptide library. The second library was generated by combining peptide blocks selected from the primary library and subjecting them to the next round of selection. The third library was constructed by pairing two peptides selected from the second library. (b) Schematic representation of cDNA display-based selection-by-function. SbF-worked r/d-IVV was an RNA/DNA-type *in vitro* virus construct augmented with the selection-by-function construct. SbF-worked dIVV could be cleaved by cathepsin E if its binding activated cathepsin E, as shown in the box.

The paired peptide library was generated by the Y-Ligation-based block shuffling (YLBS) method [16]. In brief, 10 species of DNA blocks were synthesized corresponding to the peptides that were based on information for cathepsin E-activating peptide aptamers obtained from the secondary library selection [9]. This resulted in construction of a library with about 400 different variants (the actual diversity was assumed to be much higher due to substitutions and indel mutations during DNA construct generation [17]). This DNA library was integrated into the cDNA display construct after the cDNA display procedure (Figure 1(b)).

### 2.3. Selection of Cathepsin-E-Activating Peptides

To select cathepsin-E-activating peptides, the selection-by-function method was used [14] with minor modifications. After completing the final cDNA display construct (Figure 1(b)) by transcription, puromycin-linker ligation, translation, and reverse transcription, the selection-by-function method was employed. First, paired peptide-coding DNA was incubated in Selection buffer (50 mM Tris-HCl, 100 mM NaCl, and 5 mM MgCl_2_, pH 7.4) containing 5 pmol of cathepsin E-immobilizing sepharose beads (GE Healthcare, USA) at 25°C for 10 min. Unbound DNA molecules were rapidly removed by washing with Selection buffer. DNA molecules that were bound to cathepsin E immobilizing beads were incubated at the temperature (37°C) optimum for cathepsin E proteolytic reaction. DNA molecules released from the beads were collected for the next selection step, as most could be assumed to be generated by the enhanced proteolytic reaction of cathepsin E. This procedure was repeated three times with increasing selection stringency (i.e., shorter reaction periods). The final product was subjected to cloning and sequencing to identify cathepsin E-activating peptides.

### 2.4. Cathepsin E Protease Activity Assay

Activation of cathepsin E by the selected peptides was determined as previously described [9]. The selected peptides were prepared by *in vitro* translation or chemical synthesis. A solution containing 20 nM cathepsin E was pre incubated with 20 nM of selected peptides in Selection buffer (50 mM Tris-HCl, 100 mM NaCl, pH 7.4) at 25°C for 10 min. Next, the fluorogenic substrate was added (5 *μ*M) and the mixture was incubated at 37°C for 1 h for the enzyme reaction. The fluorescent reaction product was monitored at 440 nm (excitation at 340 nm) using Infinite 200 (TECAN, Japan). The percent of cathepsin E activation (*A*) was calculated by:

(1) $$\begin{array}{c} A\;=\;100\times \frac{\left(S_{f}-B_{f}\right)}{\left(C_{f}-B_{f}\right)\;}\left[\%\;\text{activation}\right], \end{array}$$

where *S*_*f*_ was the fluorescence intensity of the cathepsin E reaction in the presence of a selected peptide, *C*_*f*_ was the fluorescence intensity of the control in the absence of a selected peptide, and *B*_*f*_ was background fluorescence of the solution containing the fluorogenic substrate only. 

### 2.5. *In Vitro* Translation or Chemical Synthesis of Selected Peptides

The selected peptides were prepared by* in vitro* translation or chemical synthesis, depending on their intended use. To rapidly estimate cathepsin E-activating capability, peptides were prepared by *in vitro* translation using the DNA coding sequence for the selected peptide. The coding sequence was integrated into the DNA construct for *in vitro* translation [8]. The *in vitro* translated products consisted of a streptavidin-binding peptide for molecular fishing, a protease Factor Xa recognition site for removing the unnecessary peptide portion, and a functional peptide coding region. Finally, peptides were synthesized using a translation kit (PURESYSTEM, Wako, Japan) following the manufacturer's protocol. Peptides T4 (IEGRGCPCIDFMVEVQVEVAEALLTALSLSPGS) and T11 (IEGRLLSGGAGACSVRTVDDSFDCG) were chemically synthesized by commercial vendors (SCRUM Corporation, Ltd., Japan and Operon Biotechnologies, Inc., Japan) with certification of more than 95% purity for the precise assays of cathepsin E activity/affinity/*in vitro* or *in vivo*.

### 2.6. Affinity Determinations

The dissociation constant (*K*_*d*_) of each selected peptide with cathepsin E was determined by the SPR method using a Biacore2000 (GE Healthcare, UK). Cathepsin E was immobilized on a CM5 Biacore sensor chip (GE Healthcare, UK) by the general amine coupling method. A small quantity of acetate buffer (50 mM sodium acetate, 100 mM NaCl, pH 4.5) containing cathepsin E (150 *μ*g/mL) was injected into the flow cell for the sample lane. The reference lane was prepared similarly but without cathepsin E. Different concentrations (10, 20, 30, and 40 nM) of the selected peptide candidates, T4 and T11 were injected into both lanes at a flow rate of 20 ul/min to measure the interaction between cathepsin E and each peptide candidate. For all experiments, a neutral pH buffer (50 mM Tris-HCl, 100 mM NaCl, pH 7.4) was used as the running buffer and a solution of 50 mM NaOH was used to remove the peptides. The resulting sensorgram curves were fit to a 1 : 1 Langmuir binding model, and the *K*_*d*_ values were calculated using BIA evaluation software (GE Healthcare, UK).

### 2.7. Cell-Based Assay

HeLa cells were used for an* in vitro* cell-based bioassay. Approximately 10,000 HeLa cells were seeded into the wells of a 96-well culture plate (Corning, USA) containing 100 uL of DMEM medium supplemented with 10% FBS and 2% penicillin-streptomycin and incubated overnight at 37°C under 5% CO_2_. The cells were treated with cathepsin E (77 nM) alone or together with two different concentrations (770 nM, 7.7 *μ*M) of the selected peptide T11 in 100 *μ*L of serum-free Opti-MEM at 37°C for 20 h. After incubation, viable cells were determined with a Cell Counting Kit-F (Dojindo Molecular Technologies, Japan) according to the manufacturer's protocol. Viable cells were identified by their fluorescent emissions intensity using an Infinite 200 (TECAN, Japan) with excitation at 490 nm and emission at 515 nm. To detect apoptotic cells, cells were treated with Annexin V-Cy3 (BioVision, USA) following the manufacturer's protocol. Apoptotic cells were detected by their fluorescent emissions with excitation at 543 nm and emission at 570 nm. To detect caspase-3/7 activity induced by cathepsin E, an Apo-ONE Homogeneous Caspase-3/7 Assay kit (Promega, USA) was used according to the manufacturer's protocol. Activity was detected by fluorescent emissions with excitation at 485 nm and emission at 530 nm. The cathepsin E and selected peptide concentrations used for apoptosis and caspase assays were the same as those used for cell viability assays.

## 3. Results

We sought to develop a method to obtain cathepsin E-activating peptides and identify a sufficiently strong enzyme activator with verifiable biological activity, such as inducing cancer cell apoptosis.

### 3.1. Overall Scheme to Obtain Activating Peptides

Cathepsin-E-activating peptides that were previously developed (Table 1) were used as starting materials for this study. These peptides moderately enhanced cathepsin-E activity (~60% using our assay system) and had a maximum binding affinity of 400 nM. These activity-enhancing peptides were selected using the selection-by-function method [17] and a block shuffling method: ASAC (all-steps all-combinations; Figure 1). Among various selection methods, the selection-by-function method was found to select activating peptides and the ASAC method provided a proven library, from which we could identify peptides with improved activities [8, 9, 18]. These methods enabled the selection of the desired peptides that otherwise would have been difficult, as there is no general approach to effectively identify protease-activating reagents (including peptides).

Thus, we introduced a novel method that exploited previously acquired molecular information, that is, pairing of selected peptides that have affinity for a target protein, specifically for different epitopes, to increase activity in a cooperative manner. The library that was generated by arbitrarily pairing the second library selection products (Table 1) was the third library (i.e., paired peptide library). This library was screened by the selection-by-function method (Table 2). For the technical reasons (see [8, 18] for details), the initial paired peptide library contained substantial amounts of unintended molecules in addition to the whole set of intended molecules.

### 3.2. Activity Enhancing Peptides Acquired by the Paired Peptide Method

**Figure 2:**
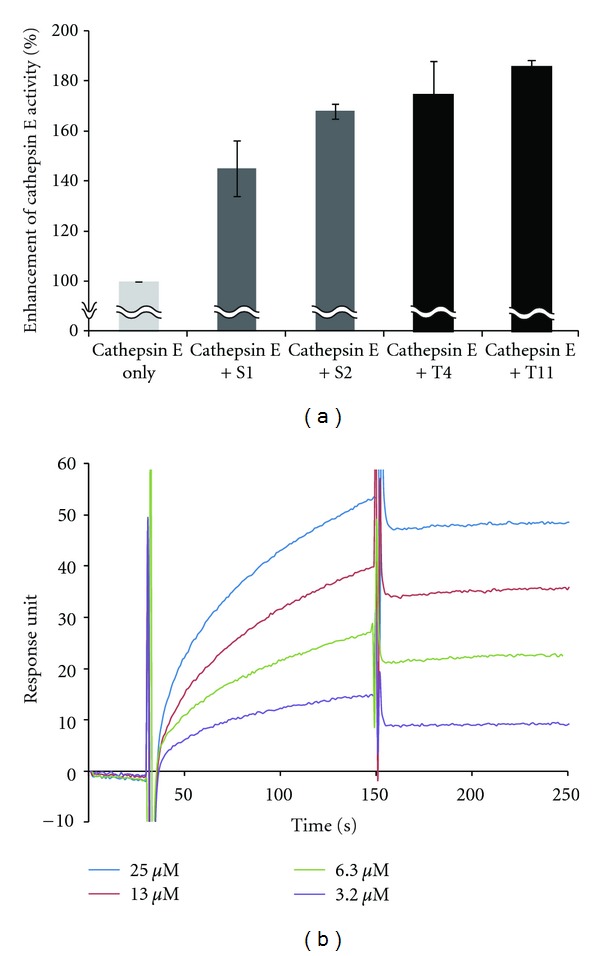
Activities and affinities of selected peptides for cathepsin E activation. (a) Cathepsin E activity enhancement by chemically synthesized peptides selected from the secondary ASAC library (S1 and S2) and the third paired peptide library (T4 and T11). Cathepsin E and peptide concentrations were 20 nM. Error bars indicate the standard deviations of triplicate experiments. (b) Typical SPR sensorgram obtained from the interaction between paired peptide T4 and cathepsin E. To determine the dissociation constant (*K*_*d*_), four different peptide concentrations were injected. The range from 40 s to 150 s corresponded to association, while that from 150 s to 250 s corresponded to dissociation.

A few selected clones were assayed for cathepsin E-activating capability using *in vitro* translation products (Supplementary Figure 1 available online at doi:10.1155/2012/316432). Two peptides selected by this method were analyzed further using chemically synthesized peptides and were found to be clearly superior with regard to their activity or binding affinity than the previously selected products (Figure 2). Peptide T11 had 1.3 times greater activity than the most improved activity peptide (S2) in the second library of selected products. Another peptide, T4, had a very high affinity for cathepsin E (*K*_*d*_ = 2 nM). 

It is worth noting that, although the affinity of peptide T11 to cathepsin E was too weak to be determined by the conventional SPR method (two failed trials), peptide T11 did have a high activating capability in a preliminary test. This situation sometimes occurs, as reported for PDK1 activators [20]. Considering that the members of the third library, including those that were unintentionally generated, were a relatively enriched population and they comprised moieties that had already been selected for their cathepsin E activation, the remarkable improvement shown here was expected. 

### 3.3. Biological Activity of a Cathepsin E Activator: Apoptosis Induction

**Figure 3:**
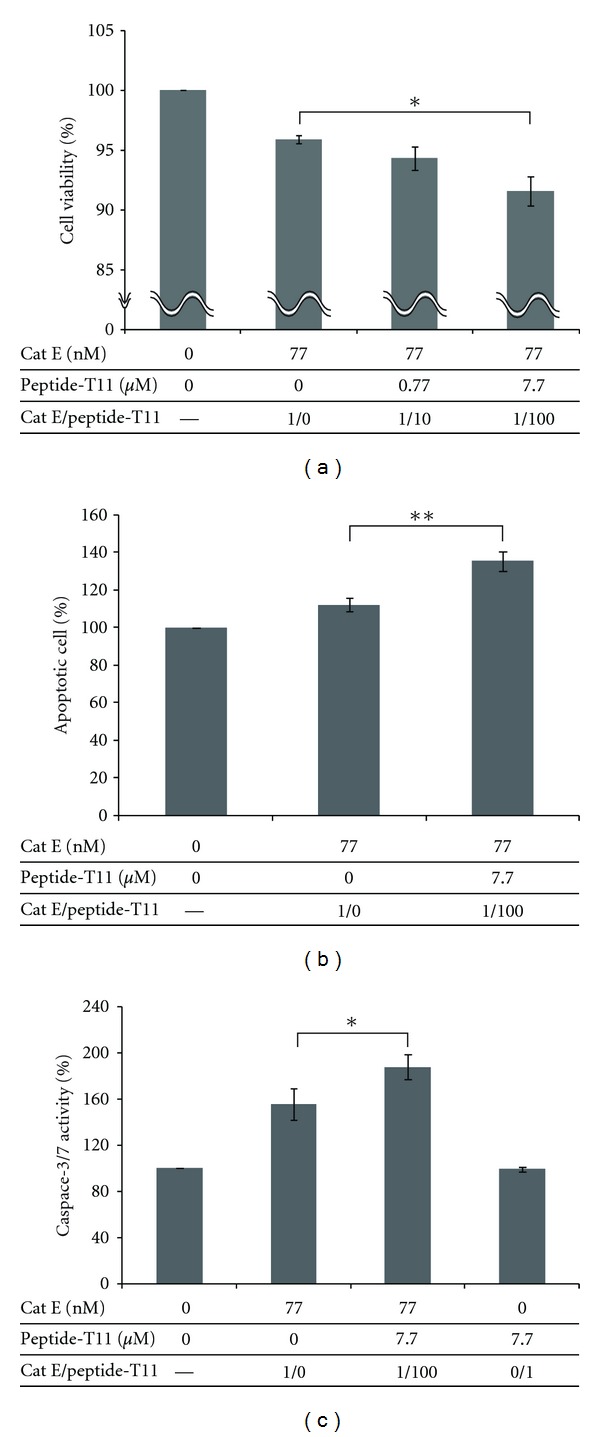
Biological effects of a paired peptide on HeLa cells. (a) Induction of cancer cell death by cathepsin E and its enhancement by a peptide. Cell viability was determined using a cell counting kit after treating HeLa cells for 20 h with cathepsin E and peptide T11 at different molar ratios (cathepsin E: peptide = 1 : 0, 1 : 10, 1 : 100). (b) Effect of a cathepsin E-activating peptide on cancer cell apoptosis. Apoptotic cells were stained with Annexin V-Cy3 for 24 h after incubating HeLa cells in the presence of 77 nM cathepsin E and 7.7 *μ*M peptide T11. (c) Assessment of caspase activity induced by cathepsin E-activating peptide. Caspase-3 and/or -7 was measured at 24 h after incubating HeLa cells in the presence of 77 nM cathepsin E and 7.7 *μ*M peptide T11. Error bars indicate the standard deviations of three independent experiments. Statistical significance is denoted by the symbols (* < 0.05, **< 0.01) and was based on comparison by Student's *t*-test.

The peptide T11 exhibited the highest cathepsin-E-activating ability; thus, we used it in an apoptosis induction assay. As shown in Figures 3(a) and 3(b), the percentage of dying cells in the presence of T11 was significantly higher than without it. This apoptosis phenomenon was confirmed by detecting of increased levels of apoptosis-associated protein caspase-3/7 (Figure 3(c)). TRAIL-induced-apoptosis, which is assumed to be the pathway responsible for cathepsin-E-mediated cell death [11], results in caspase-mediated apoptosis, including the activity of caspase-3/7. Caspase-3/7 activity was found to be activated by the addition of peptide T11 to a much higher level of apoptosis (188%) than with cathepsin E alone (155%). This phenomenon was not observed when T11 was added without cathepsin E.

Although peptide T11 could be assumed to induce cancer cell apoptosis through its activation of cathepsin E, the actual mechanism may not be so simple. It was previously proposed that cathepsin E cleaved off the soluble TRAIL ligand from the TRAIL precursor protein and that this ligand switched on the apoptosis pathway of cancer cells. There may also be another pathway for cathepsin E-induced apoptosis of cancer cells (i.e., different target for cathepsin E than the TRAIL precursor protein). Our preliminary data may support this proposition, as we attempted to find the cleaved TRAIL molecule when HeLa cells were treated with cathepsin E and the cathepsin E-activating peptide (T11). With these conditions, apoptosis could be induced, although the cathepsin E concentration was significantly lower (77 nM) than that previously reported (1 *μ*M) [11].

## 4. Discussion

In this study, we attempted to establish and confirm an approach for identifying protease-activating peptides. In general, it is more difficult to identify protease activity-enhancing molecules than activity-inhibiting molecules because no general principles have been established, whereas activity-inhibiting molecules are rather easily obtained by fabricating substrate-analog molecules. Yet, increasing the activity of a particular protease often contributes not only to elucidating molecular systems within cells, such as caspase signal transduction and metabolic pathway regulation, but can also enhance activities, such as antimicrobial or anticancer activities [21].

It is likely that diminished protease activity, which leads to the accumulation of unprocessed proteins or a shortage of necessary processed proteins, plays a causative role in various diseases. To treat these diseases, either the amount of the protease needs be increased or the protease activity needs be enhanced. The latter is much easier to accomplish because of the small size and high stability of proteases. Therefore, the aim of this study, to identify cathepsin E-activating peptides was reasonable.

 The peptide that we identified here, T11, was associated with inducting cancer cell apoptosis, as shown in Figures 3(a) and 3(b). Cancer cells apoptosis was further confirmed by increased levels of apoptosis-related factors (caspase-3 and/or -7) that were observed in the presence of cathepsin E and its activator T11 (Figure 3(c)). As expected, the peptide alone had no effect on cells. Because tumor growth was suppressed by cathepsin E treatment in a mouse xenograft model [11], T11 administration might be expected to enhance therapeutic efficacy. 

Another important finding of this study was the consistent, high performance of *in vitro* evolution reported in a previous study [9]. This was confirmed here by the observed steady improvement in activities of the selected products as the stage of the library progressed from first to second, and from second to third (Table 2 and [9]). It is worth noting that some of the mutation-derived peptides had a higher capability for cathepsin E activation than the non-mutated peptides and ultimately survived the selection-by-function processes. Because this approach could actually find more functional peptides than what was expected, this library that contained unintentionally mutated molecules was effective.

Although this demonstration was rather phenomenological and will require many confirmatory examples before it is sufficiently reliable, the present data together with previously reported data (7 trials; 4 trials regarding cathepsin E inhibition and activation at acidic pH ([8] and Kitamura et al.), [21]), 2 trials regarding cathepsin E activation at neutral pH ([9] and this study) and one about A*β*42-binding peptides [18]) strongly support the idea that the progressive library method is an effective means for identifying activity-enhancing peptides.

## 5. Conclusions

By adopting the systemic *in vitro* evolution method (eRAPANSY) augmented by function-based screening (selection-by-function), we could identify peptides that had high activating capability for the protease cathepsin E. We also demonstrated the availability of these activators for the induction of cancer cell apoptosis. The same strategy is most likely to be applicable to develop the clinically available peptide activators which are targeted to other proteases.

## Supplementary Material

Supplementary figure 1: Functional screening of cathepsin E activating peptides.The strong activators of cathepsin E were screened based on the enzyme activity assay using in vitro translated peptides. The concentration of cathepsin E and each peptide is the same (20 nM). The cathepsin E activity without any peptide was also measured as control.

## Figures and Tables

**Table 1:** Cathepsin-E-activating peptides and spacer peptides used for paired peptide library construction.

Name^1^	Size (a.a.)	Amino acid sequence (N→C)	Activity (%)^2^
S1	13	IEGRVGCDFMYVG	**130**
S2	8	GSPCIGII	*133 *
S3	8	IVIHQQLL	—
S4	8	PGIKIIIIG	*151 *
S5	9	IGPQFGMCG	—
S6	10	PGFEERSSEG	—
S7	16	SPIISHIVGCDPPSCG	*160 *
S8	16	IGCEERSFPNIIIIIG	**168**
S9	13	SGIKVGCDPPSCG	*140 *
S10	13	PGIKPPPCIIIIG	**145**
s1	10	GGGSGGGSGG	—
s2	10	GGGPGGGPGG	—
s3	15	GGGSGGGSGGGSGGG	—
s4	15	GGGPGGGPGGGPGGG	—

^1^Cathepsin E-activating peptides (S1–S10) obtained from primary library selection [9] and spacer peptides (s1–s4) are shown.

^2^Activities were determined using peptides produced by the *in vitro* translation system (in italics) or chemical synthesis (in bold). Note that the former is less reliable than the latter and sometimes exhibits a higher activity than the latter and sometimes adversely systematically [19]. Cathepsin E activity alone was considered as to be 100%.

**Table 2:** Amino acid sequences of selected peptides.

Name	Paired blocks^1^	Amino acid sequence (N→C)^2^	Size (a.a.)	Activity (%)^3^	Frequency^4^
T1	S2-(s1)-*α*	**GCPCIDFM**VEVQVEVAEA**LLTALSLSPGL** **GMTATKGEFQHTGGRY**	45	—	6
T2	S2-(s1)-*α*	**SCPCIDFM**VEVQVEVAEA**LLTALSLSPGL** **GMTATKGEFQHTGGRY**	45	—	1
T3	S2-(s1)-*α*	**SCPCIDFM**VEVQVEVAEA**LLTALSLSPGL** **GMTAT**	34	—	6
T4	S2-(s1)-*α*	**IEGRGCPCIDFM**VEVQVEVAEA**LLTALSL** **SPGS**	33	**175** (*122*)	1
T5	S2-(s1)-*α*	**SCPCIDFM**VEVQVEVAEA**LLTALSLSPGL** **GM**	32	—	1
T6	S10-(s1)-*α*	**SDDKSTTL**VEVQVEVAEE**LWRHYHYLLHG**	29	*118 *	2
T7	*β*-(s2)-*γ*	**SYKDSCI**GGRGSGGGPGG**IPGRIGYIG**	25	*111 *	1
T8	*β*-(s2)-*γ*	**NYKDSCI**GGRGSGGGPGG**IPGRIGYIG**	25	—	1
T9	*δ*	**VFVVGRSCLRLARGRVHFVSG**	21	—	1
T10	*ε*-(s1)-S2	**AVDAVL**GGDPNLGG**HSIGSCG**	21	—	1
T11	*ζ* -(s1)-S1	**IEGRLLS**GGAGACSVRT**VDDSFDCG**	26	**186** (*138*)	1

^1^See Table 1 for block names detail. Novel blocks were temporarily labeled as *α*, *β*, *γ*, *δ*, *ε*, and *ζ*.

^2^Bold regions were contained in the original blocks.

^3^Peptides for activity assay were obtained by *in vitro* translation (in italic) or chemical synthesis (in bold). In this study, the discrepancy is unexpectedly large and adverse to our previous experiences due to unknown reason [19].

^4^Copy numbers found in sequenced clones.
