# Supplementary material for: Peptide-Modulated Activity Enhancement of Acidic Protease Cathepsin E at Neutral pH
Source: Int J Pept. 2012 Dec 17;2012:316432. doi: 10.1155/2012/316432 (PMC3534310; doi:10.1155/2012/316432)
Supplement: Supplementary file 1 — Supplementary figure 1: Functional screening of cathepsin E activating peptides.The strong activators of cathepsin E were screened based on the enzyme activity assay using in vitro translated peptides. The concentration of cathepsin E and each peptide is the same (20 nM). The cathepsin E activity without any peptide was also measured as control. [file 316432.f1.pdf]

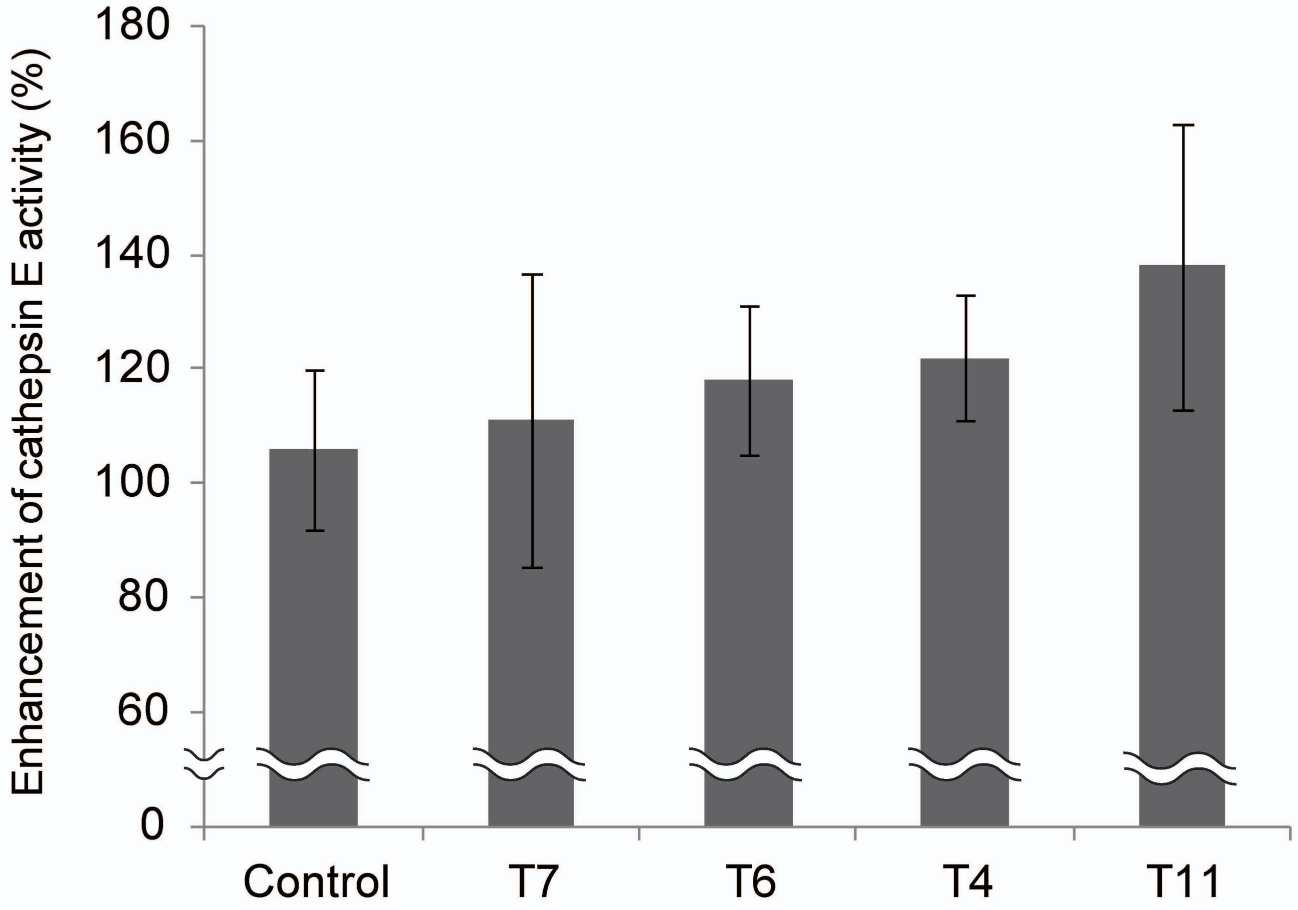

Supplementary figure 1. Functional screening of cathepsin E activating peptides.

The strong activators of cathepsin E were screened based on the enzyme activity assay using *in vitro* translated peptides. The concentration of cathepsin E and each peptide is the same (20 nM). The cathepsin E activity without any peptide was also measured as control .

Error bars represent the standard deviation of three independent experiments.
